# Lower urinary tract symptoms after laser enucleation of the prostate in patients with and without preoperative indwelling catheter

**DOI:** 10.1007/s11255-025-04948-7

**Published:** 2025-12-25

**Authors:** Cristina Cano Garcia, Fiona Schlesinger, Pia Bongardt, Maria Welte, Maximilian Filzmayer, Clara Humke, Luis A. Kluth, Philipp Mandel, Matthias Müller, Miriam Traumann, Felix K. H. Chun, Andreas Becker, Marina Kosiba

**Affiliations:** 1 Department of Urology, Goethe University Frankfurt, University Hospital Frankfurt, Theodor-Stern-Kai 7, 60590 Frankfurt Am Main, Germany; 2 Martini -Klinik, Prostate Cancer Center, University Medical Center Hamburg-Eppendorf, Hamburg, Germany; 3 Urologisches Zentrum Am Boxberg, Neunkirchen, Germany

**Keywords:** Lower urinary tract symptoms, Laser enucleation of the prostate, Preoperative indwelling catheter

## Abstract

**Purpose:**

To test for differences in outcomes of lower urinary tract symptoms (LUTS) between patients with and without preoperative indwelling catheter after laser enucleation of the prostate (LEP).

**Methods:**

In our tertiary-care database, patients undergoing LEP (11/2017–09/2023) were retrospectively analyzed, stratified by presence of preoperative catheter. Mixed linear models assessed International Prostate Symptom Score (IPSS) and quality of life (QoL) at 1, 3, 12, and 24 months after LEP. Multiple linear regression, adjusted for age and prostate volume, tested for differences in LUTS and QoL recovery.

**Results:**

Among 518 patients, 132 (25%) had a catheter before LEP (89% transurethral, 11% suprapubic), mainly due to acute urinary retention (82%). Mean catheter duration was 13 weeks; 48% exceeded 12 weeks. Catheter patients were older (71 vs. 69 years, p = 0.003), had higher PSA (8.3 vs. 5.8 ng/ml, p < 0.001), and larger prostate volume (98 vs. 86 ml, p = 0.004). At 1 month, catheter patients reported lower IPSS (7.1 vs. 9.8) and more favorable QoL (1.6 vs. 2.3). No significant differences were seen at 3, 12, and 24 months in adjusted IPSS. In regression analyses, preoperative catheter was associated with improved IPSS at 1 (coefficient -2.8, p < 0.001), 3 (-2.6, p < 0.001), and 12 months (-1.8, p = 0.007), as well as better QoL at 1 (beta -0.8, p < 0.001), 3 (-0.7, p < 0.001), and 12 months (-0.4, p = 0.01).

**Conclusion:**

Patients with preoperative catheterization appear to experience similar long-term benefits from LEP compared to those without catheter, but show greater improvements in LUTS and QoL within the first postoperative year.

**Supplementary Information:**

The online version contains supplementary material available at 10.1007/s11255-025-04948-7.

## Introduction

Benign prostatic obstruction is a common cause of lower urinary tract symptoms (LUTS) in older men. Laser enucleation of the prostate (LEP) is widely considered the preferred surgical treatment for managing benign prostatic obstruction and associated LUTS due to its demonstrated efficacy and safety [1, 2]. However, prolonged bladder outlet obstruction due to benign prostatic obstruction can cause chronic changes in the bladder, resulting in impaired contractility. This process typically progresses from detrusor hypertrophy to overactivity, followed by acute urinary retention, eventually leading to detrusor hypoactivity [3]. Preoperative indwelling catheters can be used to manage urinary retention and avoid bladder decompensation, particularly in patients with severe LUTS. However, some patients with high post-void residual volumes may decline catheterization, despite its potential benefits in preventing further bladder deterioration. There is conflicting evidence on the functional outcomes of LUTS after LEP in patients with a history of acute urinary retention or impaired bladder contractility [4–9]. Furthermore, limited data exists regarding postoperative LUTS outcomes in patients who were dependent on an indwelling catheter before undergoing LEP. Given these challenges, the relationship between preoperative indwelling catheter use and postoperative LUTS after LEP remains uncertain. To our knowledge, few studies have directly compared functional outcomes in patients undergoing LEP with and without a preoperative catheter. We hypothesize that patients with preoperative indwelling catheters may have distinct postoperative LUTS compared to those without catheters, potentially due to chronic bladder changes associated with prolonged urinary retention. This study aims to compare postoperative LUTS in patients undergoing LEP, stratified by the presence or absence of a preoperative indwelling catheter. To test this hypothesis, we relied on our institutional tertiary-care database of patients with LUTS treated with LEP.

## Subjects and methods

### Patient cohort

We studied our prospectively maintained institutional tertiary-care database to retrospectively identify consecutive patients with LUTS treated with LEP between 11/2017 and 09/2023 (N = 1145). The surgeries were performed by six surgeons, including two senior LEP experts (more than 100 surgeries), while the others reached proficiency through a structured mentoring program that was previously investigated and maintained high-quality outcomes throughout the learning curve [10]. Exclusion criteria consisted of unknown status of preoperative catheter (n = 94), unknown duration of preoperative catheter (n = 8), unknown IPSS one month after LEP (n = 469), patients with a relevant urethral stricture requiring a urethrotomy during LEP (n = 28) and patients with palliative LEP due to prostate cancer (n = 9). Although patients with postoperative catheters were excluded from the main analyses (n = 19), we reported on the rates of postoperative catheters. The final cohort for analysis consisted of 518 study patients. The study adhered to the Declaration of Helsinki, with ethical approval from the University Hospital Frankfurt's Ethical Committee and written informed consent from all participants.

### Stratification and outcomes

In this study, patients were stratified by presence or absence of a preoperative indwelling catheter before LEP; those with catheterization ≤ 1 week were classified as no catheter. Among catheterized patients, subgroups were short-term (2–4 weeks), intermediate (5–12 weeks), and long-term (> 12 weeks). The primary endpoint was IPSS, including QoL, assessed preoperatively and postoperatively via questionnaires. IPSS comprises seven items (maximum 35; mild 0–7, moderate 8–19, severe 20–35) plus an eighth QoL item (0 = excellent to 6 = very poor) [11, 12]. Secondary endpoints were IPSS storage/voiding subscores and anticholinergic treatment rates.

### Statistical analysis

The Wilcoxon rank sum and Pearson Chi-square tests examined differences in means and proportions between LEP patients, with and without an preoperative indwelling catheter, respectively. The mixed model assessed changes in IPSS and QoL at different time points after LEP. Specifically, we looked at changes 1,3, 12 and 24 after the surgery. Moreover, multiple linear regression tested for differences in patients with and without a preoperative indwelling catheter regarding IPSS und der QoL 1, 3, 12 and 24 months after LEP. Co-variables consisted of age at surgery and prostate volume (PV) measured by transrectal ultrasound. All tests were two-sided, with a significance level set at p < 0.05. R software environment for statistical computing and graphics (version 4.1.2) was used for all analyses [13].

## Results

### Baseline characteristics

Baseline characteristics are shown in Table 1. Among 518 patients, 132 (25%) had a catheter before LEP (89% transurethral, 11% suprapubic), predominantly for acute urinary retention, with a median duration of 10 weeks. Patients with a catheter were older, had higher PSA levels, and larger prostate volumes compared with those without. No differences were observed for ASA score or Clavien-Dindo complications.

**Table 1:** Baseline characteristics of 518 patients treated with laser enucleation of the prostate

Characteristic	n	Overall cohort n = 518	Indwelling preoperative catheter n = 132 (25%)	no indwelling preoperative catheter n = 386 (75%)	p-value^1^
Median age at surgery^2^	518	69 (64,77)	72 (65,77)	69 (63,74)	0.003
ASA^3^	518				0.07
I/II		387 (75%)	91 (69%)	296 (77%)	
III/IV		131 (25%)	41 (31%)	90 (23%)	
Median PSA at baseline^2^	468	4.5 (2.5, 8.3)	6.2 (3.6, 11.1)	4.0 (2.4,7.3)	< 0.001
Median PV (ml)^2^	513	80 (59,110)	87 (66,120)	79 (56,102)	0.004
PVR ≥ 200 ml^2^		–	–	72 (19%)	-
Laser^3^	518				1.0
Holmium		422 (81%)	108 (82%)	314 (81%)	
Thulium fiber laser		96 (19%)	24 (18%)	72 (19%)	
Reasons for preoperative permanent catheter^3^					
Urinary retention		–	108 (82%)	–	
High PVR		–	16 (12%)	–	
Other		–	8 (6%)	–	
Type of catheter^3^					
Transurethral		–	117 (89%)	–	
Suprapubic		–	15 (11%)	–	
Duration of preoperative catheterization (weeks)^2^			10 (4,16)	–	
Duration of preoperative catheterization – groups^3^					
Short-term (2–4 weeks)			37 (28%)	–	
Intermediate-term (5–12 weeks)			46 (35%)	–	
Long-term (> 12 weeks)			49 (37%)	–	
30 days postoperative complication (CDC grade)^3^	518				0.4
no complications		419 (81%)	107 (81%)	312 (81%)	
CDC I		28 (5%)	6 (5%)	22 (6%)	
CDC II		41 (8%)	8 (6%)	33 (9%)	
CDC IIIa		0 (0%)	0 (0%)	0 (0%)	
CDC IIIb		30 (6%)	11 (8%)	19 (5%)	
CDC IV		0 (0%)	0 (0%)	0 (0%)	
CDC V		0 (0%)	0 (0%)	0 (0%)	

^1^Wilcoxon rank sum test; Pearson's Chi-square test, 2 median (95% confidence intervall), 3 n (%)

*ASA* American Society of Anesthesiologists, *PSA* prostate-specific antigen, *PV* prostate volume (measured by transrectal ultrasound), *Qmax* maximal urinary flow rate, *PVR* post-void Residual Urine, *CDC* Clavien Dindo Classification

### Changes in IPSS in patients with vs. without preoperative indwelling catheter

**Fig. 1:**
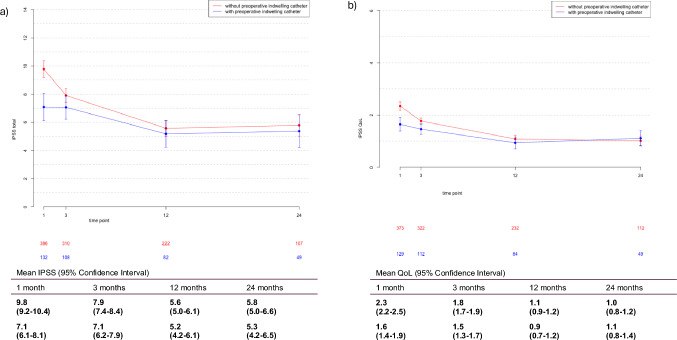
**a** Mean adjusted differences of total IPSS and **b** of IPSS-QoL according to patients with and without preoperative indwelling catheter and 95% confidence intervals 1,3, 12 and 24 months after LEP

Figure 1a shows changes in IPSS over time in patients with and without a preoperative catheter. At 1 month, catheter patients reported significantly lower scores, but no differences were observed at 3, 12, or 24 months. Similar patterns were found for storage and voiding subscores (Supplementary Figs. 1–2).

### Changes in IPSS- QoL subscore in in patients with vs. without preoperative indwelling catheter

Figure 1b shows changes in IPSS-QoL over time in patients with and without a preoperative catheter. At 1 month, catheter patients reported significantly better QoL, but no differences were observed at 3, 12, or 24 months.

### Multiple linear regression regarding IPSS

In multivariable analyses (Table 2), preoperative catheterization was associated with more favorable IPSS at 1, 3, and 12 months, but not at 24 months. Older age predicted less favorable IPSS at 1 and 12 months.

**Table 2:** Multiple linear regression testing for independent predictor status of IPSS at different time point following laser enucleation of the prostate

	1 month		3 months		12 months			24 months		
Characteristic	Beta (95% CI^1^)	p- value	Beta (95% CI^1^)	p- value	Beta (95% CI^1^)	p-value		Beta (95% CI^1^)	p-value	
Catheter status										
*no catheter*	*Reference*									
*catheter*	− 2.84 (-4.01, − 1.66)	< 0.001	− 2.61 (− 3.80, − 1.43)	< 0.001	− 1.81 (− 3.13, − 0.50)		0.007	− 1.05 (− 2.59, 0.50)		0.2
Age at surgery	0.08 (0.01, 0.14)	0.02	0.06 (− 0.01, 0.13)	0.07	0.10 (0.02, 0.17)		0.01	0.02 (− 0.07, 0.12)		0.6
Baseline PVR	− 0.003 (− 0.01, 0.009)	0.6	0.00 (− 0.01, 0.01)	1.0	− 0.01 (− 0.03, 0.001)		0.08	− 0.01 (− 0.03, 0.002)		0.1

*CI* confidence interval, *PV* prostate volume (measured by transrectal ultrasound), *Qmax* maximal urinary flow rate, *PVR* post-void residual urine, *IPSS* International Prostatic Symptom Score

### Multiple linear regression regarding QoL

In multivariable analyses (Table 3), preoperative catheterization was associated with better QoL up to 12 months, but not at 24 months. Older age predicted worse QoL at 1, 3, and 12 months.

**Table 3:** Multiple linear regression testing for independent predictor status of QoL at different time point following laser enucleation of the prostate

	1 month		3 months		12 months			24 months		
Characteristic	Beta (95% CI1)	p- value	Beta (95% CI1)	p- value	Beta (95% CI1)	p-value		Beta (95% CI1)	p-value	
Catheter status										
*No catheter*	*Reference*									
*Catheter*	-0.76 (-1.08, -0.44)	< 0.001	-0.70 (-1.00, -0.40)	< 0.001	-0.40 (-0.66, -0.13)		0.003	-0.21 (-0.55, 0.13)		0.2
Age at surgery	0.03 (0.01, 0.04)	0.003	0.02 (0.01, 0.04)	0.006	0.03 (0.01, 0.04)		0.001	0.02 (-0.001, 0.04)		0.07
Baseline PV	-0.001 (-0.004, 0.002)	0.5	0.001 (-0.002, 0.004)	0.6	-0.002 (-0.01, 0.001)		0.1	0.001 (-0.003, 0.004)		0.7

*CI* confidence interval, *PV* prostate volume (measured by transrectal ultrasound), *Qmax* maximal urinary flow rate, *PVR* post-void Residual Urine, *IPSS* International Prostatic Symptom Score

### Anticholinergic treatment after surgery

Anticholinergic treatment rates are shown in Supplementary Table 1. Three months after LEP, the rate of anticholinergic treatment was significantly lower than for patients without preoperative catheter (5.6% vs. 14.6%, p = 0.02). No significant differences in rates of anticholinergic treatment were observed at 1, 12 and 24 months after LEP.

### Postoperative catheter

Rates of postoperative catheter, excluded from the main analysis, are shown in Supplementary Table 2. One month after LEP, catheterization rates were 3.5% in patients with preoperative catheter compared to 1.8% in those without. Rates were 4.1% versus 0.6% at three months post-LEP. At 12 months, catheterization was not observed (0%) in patients with preoperative catheter, compared to 0.4% in those without. At 24 months, rates were 1.9% versus 0%.

## Discussion

We tested for differences in LUTS after LEP in patients with vs. without perioperative indwelling catheter within our institutional tertiary-care database. We made several important observations.

First, we made important observations regarding the baseline characteristics of patients undergoing LEP at our institution. In our cohort, 25% of patients had a preoperative indwelling catheter. This proportion is slightly lower than the rates reported in the literature, including a systematic review by Pallauf et al. (30.1%) and a study by Capogrosso et al. (32.1%) [7, 14]. Despite a 7% lower rate of preoperative catheterization, our cohort was otherwise comparable to that of Capogrosso et al., particularly in terms of median age (69 vs. 68 years) and prostate volume (80 vs. 80 mL) [7].

Second, we observed no significant differences in 30-day postoperative complication rates in patient with and without preoperative catheter before LEP. This finding contrasts with the results by Capogrosso et al., who reported that the presence of preoperative catheter was associated with an increased risk of significant postoperative complications defined as Clavien-Dindo classification (CDC) ≥ 2 [7]. Similarly, Denimal et al. reported a 50% complication rate in patients undergoing surgery for benign prostatic hyperplasia with preoperative indwelling catheter [6]. These rates are noticeably higher than those observed in our cohort focusing on patients with preoperative catheter, where the overall complication rate across all grades of the CDC was 19% (CDC grade I: 5%; CDC grade II: 6%; CDC grade III: 8%). It is of note that patients in our cohort were slightly younger (median age 72 vs. 75 years) but presented with higher prostate volumes (median 87ml vs. 78ml) compared to the cohort by Denima et al.

Third, we made important observations regarding LUTS following LEP. One month after LEP, patients with preoperative indwelling catheter reported better LUTS outcomes, reflected by lower IPSS scores compared to those without preoperative catheter (mean IPSS 7.1 vs. 9.8). Moreover, preoperative catheter status was a significant predictor of lower IPSS at 1, 3, and 12 months post-LEP in multiple regression analyses. These findings are in contrast to the results reported by Yuk et al., who observed comparable IPSS scores at 3 and 6 months after LEP when comparing patients with acute urinary retention, chronic urinary retention, and no urinary retention [15]. However, it is important to note that in the study by Yuk et al., only approximately 30% of patients with either acute or chronic urinary retention had an indwelling catheter. In contrast, Presicce et al. identified acute urinary retentions and increased PVR as factors associated with less favorable surgical outcomes, especially in patients treated with TURP [16]. Similarly, Ahmed et al. reported worse outcomes in patients undergoing TURP with acute urinary retention compared to patients without [17]. However, Ahmed et al. did not specify the proportion of patients with preoperative catheters within the acute urinary retention group. On the other side, Frendl et al. reported poorer long-term postoperative outcomes in patients with acute urinary retention and history of catheterization [16, 18]. However, these studies are not directly comparable to our study since only patients treated with LEP and not TURP were included in our study. Moreover, when looking at the IPSS voiding and IPSS storage subscores separately, we could see the same trend that 1 month after LEP, patients with preoperative catheter reported lower IPSS voiding (2.1 vs. 3.2) and IPSS storage (5.0 vs. 6.6) scores compared to patients without catheter. No difference was reported at 3, 12 and 24 months after LEP. At this point, it is important to mention that at 3 months post-LEP anticholinergic medication use was significantly higher in patients without a preoperative catheter (14.6% vs. 5.6%, p = 0.02), and showed a trend towards higher usage at 1 month as well (9.7% vs. 3.8%, p = 0.06). This may indicate a degree of bladder dysfunction in patients without prior catheterization, possibly due to decompensated bladder function from prolonged bladder outlet obstruction. Possibly supporting this, among patients without a perioperative catheter, 19% had a post-void residual (PVR) volume of ≥ 200 mL. Although there is no universal threshold for defining chronic urinary retention, the American Urological Association (AUA) suggests a PVR of > 300 mL. To minimize the risk of chronic urinary retention and its associated complications, we recommend the use of an indwelling catheter when the PVR reaches approximately 200 mL. In some cases of recurrent infections or severe symptoms we even recommend the use of an indwelling catheter with a OVR < 200 ml. Interestingly, Lopategui et al. investigated the outcomes after LEP across different PVR groups, specifically 0-100ml, 101-300ml, 301-600ml and > 600ml, and found no significant differences between these four different groups [4]. However, while their study included asymptomatic patients with preoperative catheters, the exact proportion of such patients was not specified. Additionally, Lopategui et al. did not report the duration of catheterization, a factor that may influence outcomes [4]. In our study, the median duration of indwelling catheter use was 10 weeks and 37% of patients with preoperative indwelling catheter had the catheter longer than 12 weeks. When patients require a preoperative catheter, we routinely recommend the use of a valve to maintain bladder capacity and prevent compromised storage function. Reasons for prolonged catheterization are often multifactorial, including reduced operating room capacity and longer waiting times, particularly for non-oncological urological procedures [19]. Moreover, as a university clinic, we frequently manage older patients and those with multiple comorbidities. In some instances, urgent interventions, such as cardiac surgeries, take precedence over LEP, necessitating extended catheter use. In these cases, it is crucial to actively manage urinary retention during the waiting period to reduce potential impairments in bladder health.

Fourth, although not part of the primary analysis, we reported postoperative catheterization rates following LEP. These rates were low in patients with and without a preoperative catheter, suggesting LEP may be effective in eliminating catheter dependency. Moreover, a preoperative catheter was associated with better QoL outcomes at 1, 3, and 12 months following LEP. Catheter removal likely played a key role in improving quality of life. Notably, patients with a preoperative catheter in our cohort were generally older. This highlights the potential of LEP as a treatment option even in elderly patients, who might otherwise be managed conservatively. Urologists may therefore re-evaluate surgical intervention for older patients with long-term catheterization. These findings are supported by Klein et al., who reported similar outcomes in patients aged over 85 years with preoperative catheters undergoing LEP. At 12 months, 97% of these patients no longer required catheterization.

Despite its novelty, the current study has several limitations. First, due to the retrospective design of the current study, follow-up at all time points was not available for the entire study cohort. Moreover, the retrospective nature of the study could have resulted in incomplete documentation or reporting bias that may have affected the capture of postoperative complications, potentially resulting in an underestimation of complication rates. Second, study outcomes were based on the IPSS, a patient-reported outcome measure and therefore inherently subjective. Differences in preoperative voiding status, such as the presence of an indwelling catheter, may have influenced postoperative IPSS scores. Third, detailed documentation of catheter management, whether via a valve system or continuous drainage with a collection bag, was lacking. This may have influenced bladder capacity and function. However, study outcomes relied on subjective patient-reported outcome measures and not on objective measures such as urodynamic parameters.

## Conclusion

Patients with preoperative catheterization appear to experience similar long-term benefits from LEP compared to those without catheter, but show greater improvements in LUTS and QoL within the first postoperative year.

## Supplementary Information

Below is the link to the electronic supplementary material.

Supplementary file1 (PDF 119 KB)

## Data Availability

All data generated or analyzed during this study are included in this article. Further enquiries can be directed to the corresponding author.
