# Supplementary material for: Lower urinary tract symptoms after laser enucleation of the prostate in patients with and without preoperative indwelling catheter
Source: Int Urol Nephrol. 2025 Dec 25;58(7):2421–7. doi: 10.1007/s11255-025-04948-7 (PMC13309486; doi:10.1007/s11255-025-04948-7)

Supplementary Figure 1: Mean adjusted differences of a) IPSS-storage subscore and b) IPSS-voiding subscore according to patients with and without preoperative indwelling catheter and 95% confidence intervals 1,3, 12 and 24 months after LEP.

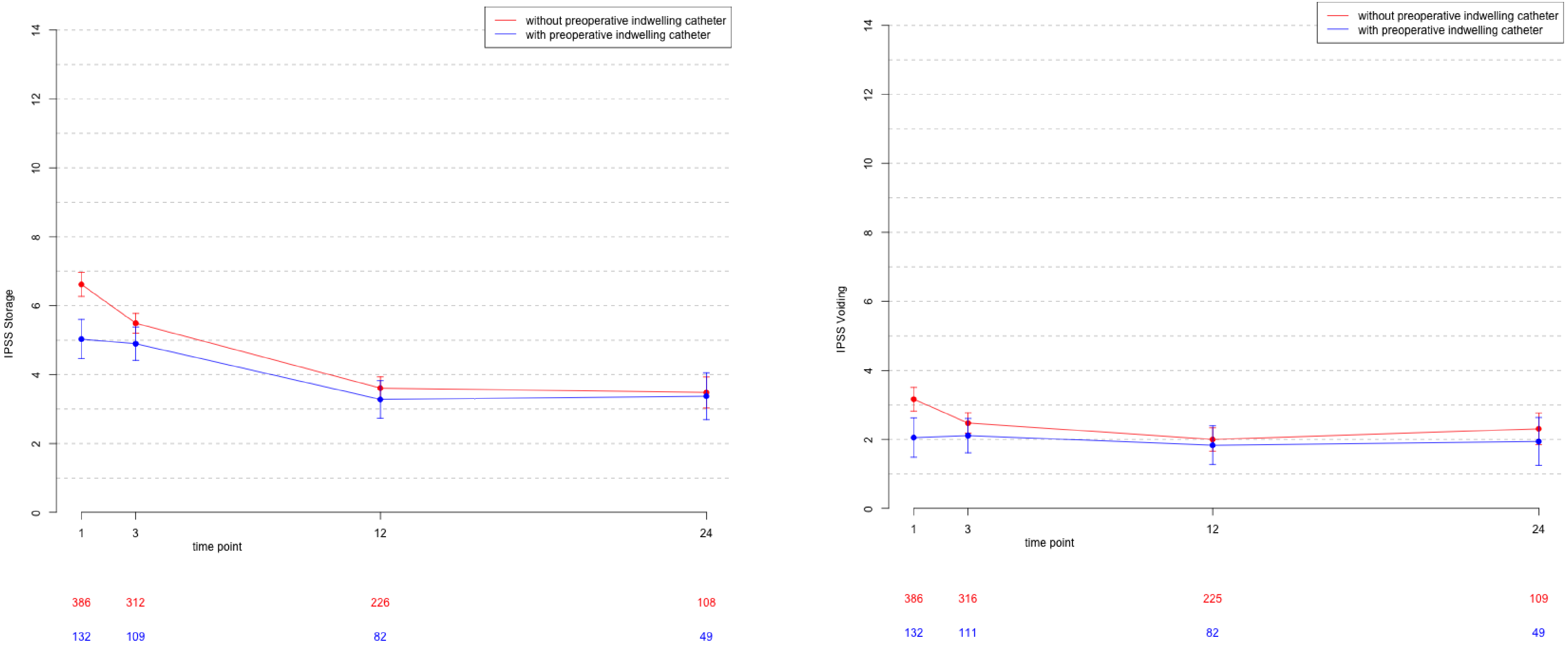

Supplement: Supplementary file 1 — Supplementary file1 (PDF 119 KB) [file 11255_2025_4948_MOESM1_ESM.pdf]
